# Modeling and Prediction of a Guidewire's Reachable Workspace and Deliverable Forces

**DOI:** 10.1109/OJEMB.2022.3233778

**Published:** 2023-01-06

**Authors:** Afsoon Nejati-Aghdam, M. Ali Tavallaei

**Affiliations:** Medical Devices and Systems LabToronto Metropolitan University 7984 Toronto ON M5B 2K3 Canada; Electrical, Computer, and Biomedical Engineering DepartmentToronto Metropolitan University 7984 Toronto ON M5B 2K3 Canada

**Keywords:** Endovascular revascularization, guide-wire, arterial occlusion, device performance, procedure planning

## Abstract

*Goal:* In this study, we investigated the feasibility of predicting the performance of a specific guidewire in terms of its ability to cover a lesion cap surface and apply force to the lesion for a given patient's vessel anatomy. The aim of this research was to provide information that could be used to plan occlusion crossings and peripheral revascularization procedures preoperatively in a way that reduces the risk of potential intraoperative complications and increases the likelihood of success. *Methods:* We used finite element (FE) analysis to simulate the interaction between the guidewire and a model of a tortuous vessel, and we used this simulation to predict the reachable workspace and deliverable forces of the device for various entry positions and angles. We then validated these predictions through experiments in which we advanced a guidewire through an identical vessel phantom using a robotic manipulator. *Conclusions:* Our findings suggest that it may be possible to predict the performance of a guidewire and forecast the likelihood of success or failure for a given vessel anatomy and lesion morphology, which could enable improved planning and device selection.

## I. Introduction

In North America, peripheral arterial disease (PAD) affects 2–14% of the general population, and its prevalence increases with age, affecting up to 29% of the elderly [1], [2]. It is estimated that the incidence of PAD is 1.6% among people aged 55–74 years [3], and unfortunately, PAD is associated with significant morbidity and mortality [4]. The main surgical procedure for treatment and management of PAD is through endovascular revascularization. Minimally invasive endovascular procedures are a preferred approach for revascularization of diseased arteries as they are less invasive, and have a lower risk for complications and morbidity in comparison to conventional bypass surgeries [5]. According to the authors [6], the guidewire is a crucial tool in endovascular treatment of arterial obstructions in lower extremity arteries and severe obstructive atherosclerosis, known as critical limb ischemia [7]. A key element in successful arterial revascularization is the successful crossing of the occlusion using the guidewire, followed by the advancement of devices such as balloons and stents beyond the lesion [6]. The success rate of crossing a chronic total occlusion (CTO) depends on various factors, including the characteristics of the CTO (such as age, length, tortuosity, cap morphology, and extent of calcification) [8], [9], [10], the device [7], [8], [11], [12], and the technique used (intraluminal versus subintimal, antegrade versus retrograde recanalization) [8], [10], [12]. In the context of our research, we are particularly interested in the role of the user/surgeon's technique in crossing heavily calcified CTOs with stiff guidewires. Therefore, it is necessary to apply a sufficiently high penetration force at the site of occlusion, particularly for highly resistant proximal caps [13]. Intraluminal crossing, although preferred to facilitate the traversal of CTOs through the lumen [14], is associated with potential challenges. The guidewire is a long and flexible device that tends to rest against the vessel wall, particularly when applying force, as the main shaft of the guidewire tends to buckle [8]. Furthermore, the procedure is guided with 2D X-ray imaging that does not provide depth perception and the user is unable to identify whether the guidewire is directed towards the lesion center or if it is at the periphery of the artery and tending to advance subintimally. When an interventionalist predicts a subintimal crossing, they may retract the guidewire, reorient it and re-navigate it in hopes of more central alignment with the lesion cap for an intraluminal crossing. However, many times this is not easily achieved and leads to unintended subintimal crossing, complications or procedure failure. This is despite the fact that peripheral CTOs have a heterogeneous morphology that includes penetrable sections at the lumen cap, such as soft tissue or microchannels [1], [15]. It would be of immense value if the interventionalist is able to know in advance or reliably predict a guidewire's reachable workspace, and deliverable forces for a given patient anatomy. Such knowledge would help avoid a trial-and-error approach and to better plan for the procedure, or device selection, to ensure proper positioning of the device at the penetrable sites of the lesion caps for true lumen crossing. While in previous work [16], knowledge of lesion morphology for prediction of device performance has been shown, that work did not consider the role of the vessel anatomy on the reachable sections of the lesion and the deliverable forces. While the above-mentioned works and many other studies in the literature have emphasized the importance of the user's experience as a key factor in the successful crossing of CTOs, the user's role is limited to adhering to established techniques. In other words, to the best of our knowledge, the additional degrees of freedom available to the user, including the insertion position and orientation of the device at the entry point and prior to the intervention for a given device and patient vascular anatomy, have not been studied in the context of CTO crossing. This is an important area of investigation that has not yet been fully explored. It is hypothesized that the reachable workspace and deliverable force of the guidewire at the occlusion site can be predicted based on the known arterial anatomy, and for various positions and orientations of guidewire insertions. These factors are likely to have a significant impact on the success rate of occlusion crossing and peripheral revascularization procedures.

## II. Materials and Methods

The position and entry angle of the guidewire into the vessel are considered as the degrees of freedom available to the physician. In this study, we will use FE simulations and experiments to determine what role these variables play in the device's reachable workspace and deliverable forces for a given patient anatomy and device characteristics.

**Fig. 1.**
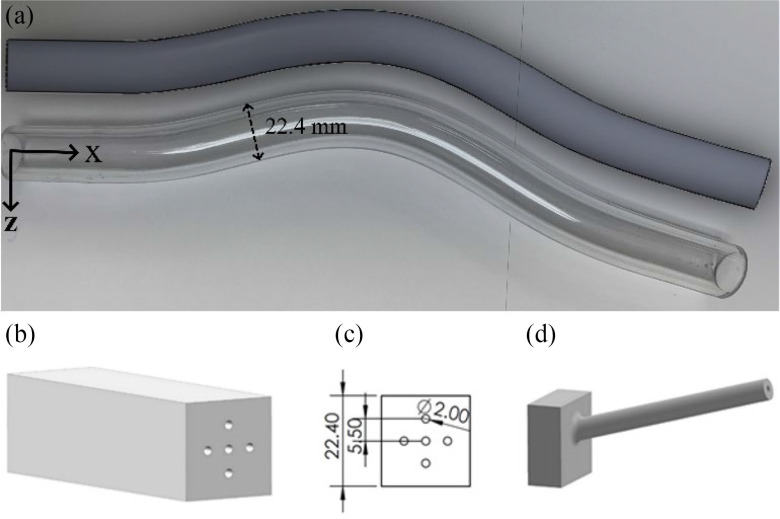
(a) Design of the artery model (top) used for the simulations and the physical artery phantom model (bottom) used for the validation experiments; CAD models of the introducer sheaths are shown in (b) the fixture with the five entry sites with insertions perpendicular to the vessel cross-section (c) the front view of the fixture shown in (b) With dimensions, (d) a representative introducer sheath model for oriented insertions.

For the remainder of the paper, assume a base reference frame $( {x,y,z} )$ (Fig. 1(a)) with the $x$-axis perpendicular to the cross-sectional area of the vessel opening. The guidewire insertion direction is aligned along the $x$-axis at the base of the phantom vessel. As for the two major axes of the cross-section of the phantom vessel at its base, the $y$-axis is chosen to point upward and be perpendicular to the plane on which the phantom vessel is located. Therefore, the phantom artery has a planar curvature in the $x - z$ plane.

### A. Finite Element Modelling-Rigid Artery Model

The motion of the guidewire and, in particular, its interaction with the vessel walls were simulated with FE analysis using ANSYS LS-DYNA (Livermore Software Technology Corporation, CA, USA) on a computer equipped with an 8-core intel i9-11900H CPU (2.5 GHz) processor. An explicit FE solver to allow more efficient simulations with complex contacts and geometric nonlinearities was adapted [17], [18].

#### 1) Geometry, Mesh, and Materials

*Guidewire:* To characterize the guidewire, our simulations use a linear elastic material model [19] with the properties given in Table I. The geometry of the guidewire is assumed to be straight wire and is modeled by serially connected beam elements with uniform circular cross-sections [20]. A model with beam elements, each with a length of 0.7 mm and a diameter of 0.89 mm, is used for the guidewire. Moreover, the Hughes-Liu with cross-section integration is chosen for the beam element formulation [17].

**TABLE I:** Material Properties of the Different Components Used in the FE Simulations

component	Young's Modulus $( {GPA} )$	Poisson ratio	Density $( {g/c{m}^{ - 3}} )$
rigid artery	3.1	0.37	1.18
rigid plaque	2.8	0.36	1.24
guidewire	21.5	0.33	6.45
sheath	2.38	0.38	1.22
deformable artery	0.17e-3	0.49	1
deformable plaque	0.04	0.4	1.45

*Artery:* In our simulations, a rigid curved tube model of 16.1 mm inner diameter (I.D.) (Fig. 1(a)) is used to simulate the occluded artery. With this rigid model, the effects of blood pressure and other physiological parameters that might affect guidewire movement are excluded and the focus is merely on the effects of geometry in our analysis. To provide consistency between the simulations and the experiments, the rigid curve is created to be identical to the actual. Fig. 1(a) compares the real phantom with its simulated counterpart. The 3D CAD model of the rigid artery is discretized into 6/8-node solid elements with a characteristic size of 1.5 mm. The corresponding material properties for the artery model (made of acrylic) are summarized in Table I [21].

*Plaque:* The plaque, or occlusion lesion, is modeled as a rigid cap at the end of the artery with 6/8-node solid elements with a characteristic size of 1 mm. The material properties are summarized in Table I and selected according to available literatures [22], to match the phantom plaque (made of PLA) used in the experiments. It can be observed that the plaque surface of the phantom vessel is tilted. Therefore, for the simulations to match the real experiments, the inclination of the plaque was measured as two independent orientations of 3.5 and 15 degrees about the two major axes of the phantom cross-section and then included in the simulations.

*Introducer Sheath:* A cylindrical rigid shell model with a diameter of 2 mm and shell thickness of 0.5 mm with quadrilateral elements is used to mimic a 6-Fr introducer [23]. The guidewire is assumed to be concentric to the sheath. Multiple different positions and angles were assumed for the introducer sheath as will be discussed in the next section. The introducer sheath position and angles are fixed during the simulation. The material properties of the simulated sheath are summarized in Table I.

#### 2) Loading and Boundary Conditions

In order to simulate the user's input during the procedure, a constant-velocity motion was defined as the user input motion and applied to the proximal end of the guidewire. To accurately reflect the clinical scenario in which the proximal end of the guidewire is held stationary by the operator's fingers, the beam element in question was fixed in all directions except for the insertion direction during the simulation. An insertion velocity of 300 mm/s was used for all simulations [24].

#### 3) Tool-Vessel Interaction

In our simulations, for modelling the contact between the rigid artery and the flexible guidewire, an automatic beam-to-surface contact detection algorithm was chosen. We performed the simulations with static and dynamic friction coefficients of 0.1 and 0.09, respectively [24] between the guidewire and vessel.

### B. Finite Element Modelling-Elastic Artery Model

In this study, we also sought to understand the contact forces between the guidewire tip and the vessel wall along the length of the artery, as this information could help us to understand the likelihood of vessel perforation during the procedure. Additionally, we were interested in how the interactions between the flexible guidewire and an elastic arterial model might affect the shape of the guidewire, its deliverable forces, and its reachable workspace. To this end, we conducted a similar analysis in Section IV using a deformable artery and plaque model to verify the accuracy of our predictions on a more realistic platform and to study the effects of vessel deformation and the potential for perforation. We used the same geometry models as in the previous simulations, but with different characteristics, for the artery and plaque, in order to establish a possible analogy with the results obtained from the rigid simulations.

As described by Roy et al. [3], the force required to puncture soft and hard lesions differs significantly. Accordingly, in this study, the plaque parameters are chosen to fit a hard lesion. Both the deformable artery and the plaque are modeled with linear elastic materials and are discretized by triangular shell elements with a characteristic size of 0.7 mm [17], using shell element formulation 4 (C0 triangular), as recommended [25]. The arterial wall and plaque are assumed to be 0.92 mm [26], and 0.5 mm thick, respectively. As for the material properties of the artery, Young's modulus of the artery is set to 0.17 MPA [26], [27], taking into account the effect of blood pressure on the vessel wall. The density and Poisson's ratio of the artery are set to 1 $( \text{g/cm}^{ - 3} )$ and 0.49 [27] respectively. The Poisson's ratio, Young's modulus, and density of the hard plaque are set to 0.4, 40 MPA [18], and 1.45 $( \text{g/cm}^{ - 3} )$ [28], respectively. Similar to [17], global damping (%15 of critical damping) is applied to simulate the surrounding tissue and structures. As discussed in [29], the geometry of the plaque is another important factor to consider in the simulations. However, to be consistent with the rest of our simulations, we assumed a flat cap for the plaque. The static and dynamic friction coefficients are set to 0.05 and 0.01, respectively [24]. In all simulations, both the distal and proximal ends of the deformable artery are rigidly fixed. We performed different simulations for the same sheath and guidewire model properties with the deformable artery and plaque model properties (Table I).

## III. Experimental Setup

**Fig. 2.**
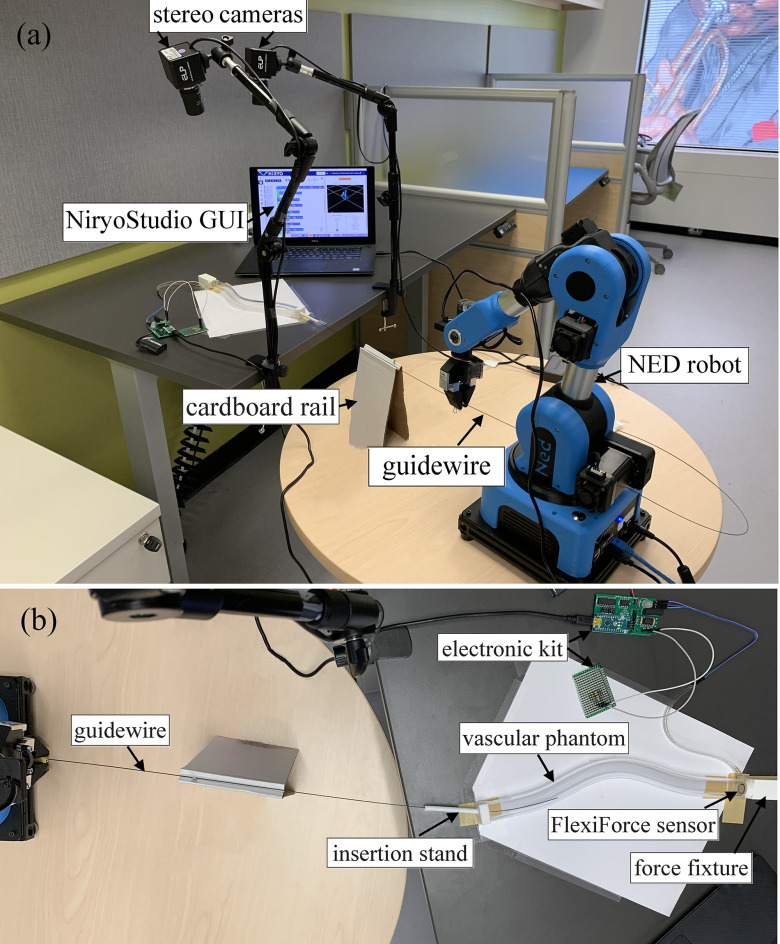
The setup used in our experiments consists of several components: (a) the guidewire, the stereovision cameras, the NED robot, the cardboard rail, and the NiryoStudio interface to control the robot; and (b) the vascular phantom, the FlexiForce sensor and its electronic kit, the insertion stand, and the force fixture.

Fig. 2 shows various components of our experimental setup, which are described below.

*Phantom artery model:* To make the phantom vessel, we used a plexiglass tube with outer and inner diameters of 22.4 and 16.1 mm, respectively (Fig. 1(a)). We then manually bent and shaped it to resemble the shape of the superficial femoral artery (SFA) (Figs. 5–6 from [30]). The average curvature of the phantom vessel is about 5 × 10^−3^ mm^−1^, consistent with the available literature [30]. To maintain a constant cross-section diameter along the entire length of the tube during manual deformation, a silicone rod with a diameter near to that of the I.D. of the tube was first inserted into the tube [31].

*Guidewire:* As recommended by the authors [6], [23], [32] a stiff 0.035-inch guidewire is the right choice, especially for crossing the lower extremity arteries. Although we chose a stiff 0.035-inch TERUMO guidewire (Terumo Corporation, Tokyo, Japan) with Radifocus J-tip, our experiments used its stiff end as a straight-tipped guidewire to simplify the modelling efforts and experimental validation. No plastic deformation of the guidewire was observed throughout the experiments.

*Robotic platform:* To provide precise and repeatable controlled motion profiles for the advancement of the guidewire, a 6-axis NED robotic arm [33] was used for this purpose. The guidewire was placed in the robot's gripper, and the NED robot was controlled with the manufacturer's software NiryoStudio.

*Stereovision system:* In our experimental setup, we used a stereovision system consisting of two cameras with varifocal lenses (5–50 mm) to track the 3D position of the guidewire tip as it was advanced through a transparent vessel phantom. The cameras (ELP 4K webcam, China) provided images with a resolution of 3840 × 2160 pixels. Prior to the experiments, the cameras were calibrated using the integrated Stereo Camera Calibrator Application in MATLAB 2020b (The Mathworks, Natick, MA) to achieve an accuracy of 0.3 mm within our desired field of view.

*Force sensor:* To enable measurement of the deliverable forces by the guidewire tip, we used a flexible piezoresistive force sensor FlexiForce A201 (Tekscan Inc., USA) [34] which measures normal compressive forces. An electronic kit (FlexiForce Prototyping Kit; Tekscan Inc., USA) was used to enable real-time logging of the measured force data on a computer.

*Introducer Sheath:* For the guidewire, we considered five different entry locations into the vessel cross-section. These entry points are referred to as the: center $( C )$, left $( L )$, right $( R )$, up $( U )$, and down $( D )$ positions, as shown in Fig. 1(b). The center entry point corresponds to the centerline of the phantom vessel cross-section at its base. For each of the five entry point locations, in order to study the effect of the guidewire orientation, we developed fixtures that allow $ \pm 15^\circ $ in up/down and left/right angles with respect to the perpendicular axis to the vessel cross-section (for brevity, only one fixture is shown in Fig. 1(d)). We refer to these as ${R}_y( { \pm 15^\circ } )$ and ${R}_z( { \pm 15^\circ } )$. Therefore, we had both perpendicular insertions as well as four orientation angles for each entry point.

*Summary of the experimental procedure:* We performed two groups of experiments in which the guidewire is robotically controlled within the phantom vessel to measure the guidewire tip's position or its contact force with the plaque. In each group, we studied a set of 25 different insertion scenarios. The first group includes experiments in which the position of the guidewire tip was tracked throughout the phantom vessel. First, the guidewire was manually inserted into the introducer sheath to ensure that its tip was seated at the opening of the introducer channel. Then, each experiment was initiated by the robot NED by executing a series of successive motion commands, each 3 mm in length, toward the introducer sheath. At the end of each motion command, the robot executed a 7-s pause command to allow sufficient time for the stereo imaging system to acquire the 3D position of the guidewire tip. To prevent the guidewire from buckling before it was inserted into the vessel phantom, we used a cardboard rail with a V-shaped groove (Fig. 2). After the guidewire made contact with the lesion cap at the end of the vessel, it was advanced an additional 1.5 cm. Each case scenario was performed only once. In the second group of experiments, we measured the contact force of the guidewire tip with the plaque. For each experiment, the guidewire was manually inserted into the introducer sheath and advanced until the distance between the tip and the plaque surface was 1 cm. The experiment was then initiated using a robotic motion command of 2.5 cm. The force data were recorded during the contact of the guidewire tip with the plaque, and the maximum force data were extracted from each experiment. In contrast to the first group of experiments, each insertion scenario was repeated forty times in this group.

## IV. Results

The results of this study were grouped into two categories. In Subsection A, we examined the effect of the *position* of the guidewire insertion (relative to the cross-section of the vessel) on the reachable space and the contact force of the guidewire tip at the occlusion site. Therefore, the results refer to the perpendicular insertions of the guidewire at different entry positions. In Section B, we looked at how the *orientation* of the guidewire at different insertion positions affected the reachable space and the contact force of the device. Subsections C and D provide a similar simulation analysis, but using deformable artery-plaque models.

### A. Influence of Guidewire Entry Location-Rigid Model

**Fig. 3.**
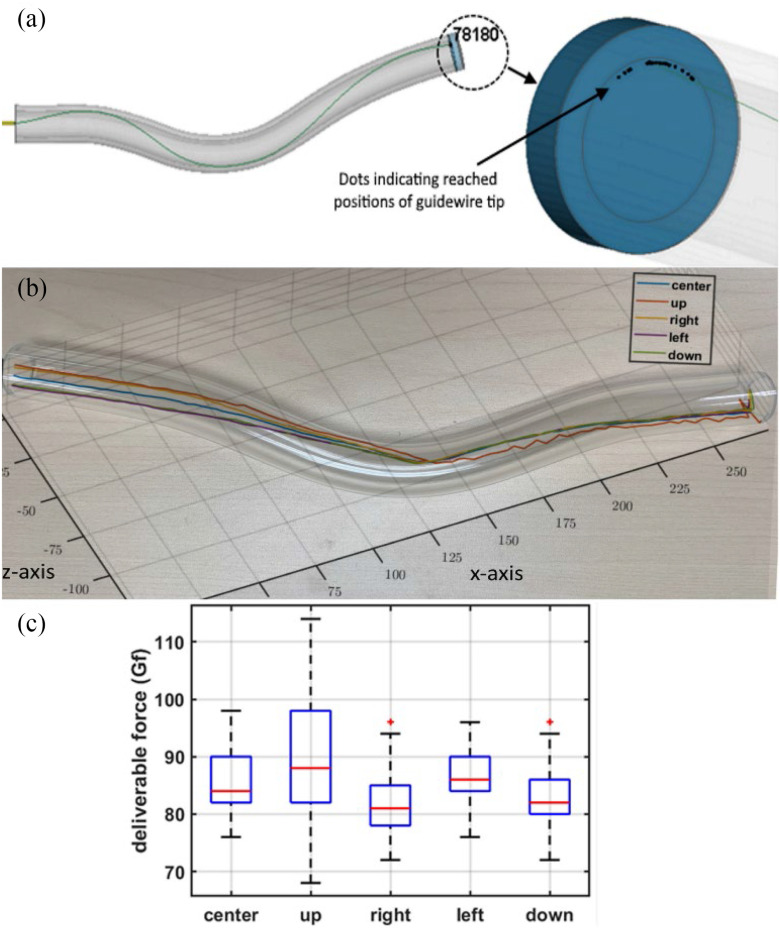
Effect of guidewire entry point, (a) Simulated results of guidewire tip position (node 78180) on the lesion cap for different insertion scenarios. The rigid artery is shown translucent to distinguish the inner and outer shells. For the center insertion point, without rotation, the guidewire is also shown, (b) Experimental force measurements at the guidewire tip for different guidewire entry points. For each entry point, the results refer to forty independent but similar test scenarios, (c) Experimental results of the 3D guidewire tip position measurements with the stereovision system for five different entry positions.

An important finding in our study was that both the simulations and the verification experiments consistently showed that the guidewire could only reach the periphery of the lesion, regardless of the entry position. Fig. 3(a) shows the simulation results of the position of the guidewire tip at the end of its path for different entry sites and orientations, while Fig. 3(b) shows the results of the 3D position reconstruction of the guidewire tip obtained using the stereovision for different entry sites of the guidewire.

As for the forces exerted by the guidewire tip on the lesion cap for different insertion points, the simulation results for the center, up, right, left, and down positions were 128, 160.8, 115.1, 125.3, and 122 gf (gram-force), respectively. Except for the up insertion point, repositioning the guidewire at different insertion points did not significantly affect the force of the guidewire tip applied to the plaque. The corresponding experimental results are shown in Fig. 3(c). A total of 40 experiments were performed for each insertion point. Table II also contains a statistical analysis of variance (ANOVA) of the measured force data set from Fig. 3(c). The p-value in each column of Table II represents the effect of changing the insertion point of the guidewire. For example, the ${C}_0 - {U}_0$ column states that by changing the guidewire insertion position from center to up, there is an insignificant ($p < 0.9954$) increase of 4.05 gf in the mean force value of the guidewire tip. The results shown in Fig. 3(c) and the information from the statistical analysis in Table II demonstrate no significant change in the force of the guidewire tip at the occlusion site due to the change in the insertion point of the guidewire.

**TABLE II:** Statistical Results of the Experimental Guidewire Tip Force for Different Insertion Points (No Rotation is Applied)

	${C}_0 - {U}_0{C}_0 - {U}_0{C}_0{U}_00$	${C}_0 - {R}_0$	${C}_0 - {L}_0$	${C}_0 - {D}_0$	${U}_0 - {R}_0$	${U}_0 - {L}_0$	$\ {U}_0 - {D}_0$	$\ {R}_0 - {L}_0$	${R}_0 - {D}_0$	${L}_0 - {D}_0$
p-value	0.9954	0.9995	>0.9999	>0.9999	0.2543	>0.9999	0.6336	0.9818	>0.9999	0.9999
Mean diff. (gf)	4.050	−3.500	1.000	−2.250	−7.550	−3.050	−6.300	4.500	1.250	−3.250

Although several shortcomings in the modelling and experimental setup may have led to the discrepancy between the simulation and experimental results, the trend seems to be similar in terms of the insensitivity of the efficiency of the revascularization procedure to different guidewire insertion points.

### B. Influence of Guidewire Entry Orientation-Rigid Model

Similar to Subsection A, the guidewire's reachable workspace is limited to the periphery of the artery at the site of occlusion regardless of the location of entry and its orientation. In other words, for the given artery shape model, the guidewire does not have access to the intraplaque area as is illustrated in Fig. 3(a). It is worth noting that Fig. 3(a) includes the results of all 25 insertion scenarios with the various positions and entry orientations. The reason that all guidewire tip positions are accumulated in a specific area of the plaque periphery instead of being distributed over the entire periphery is that the plaque surface of the phantom vessel is tilted.

**Fig. 4.**
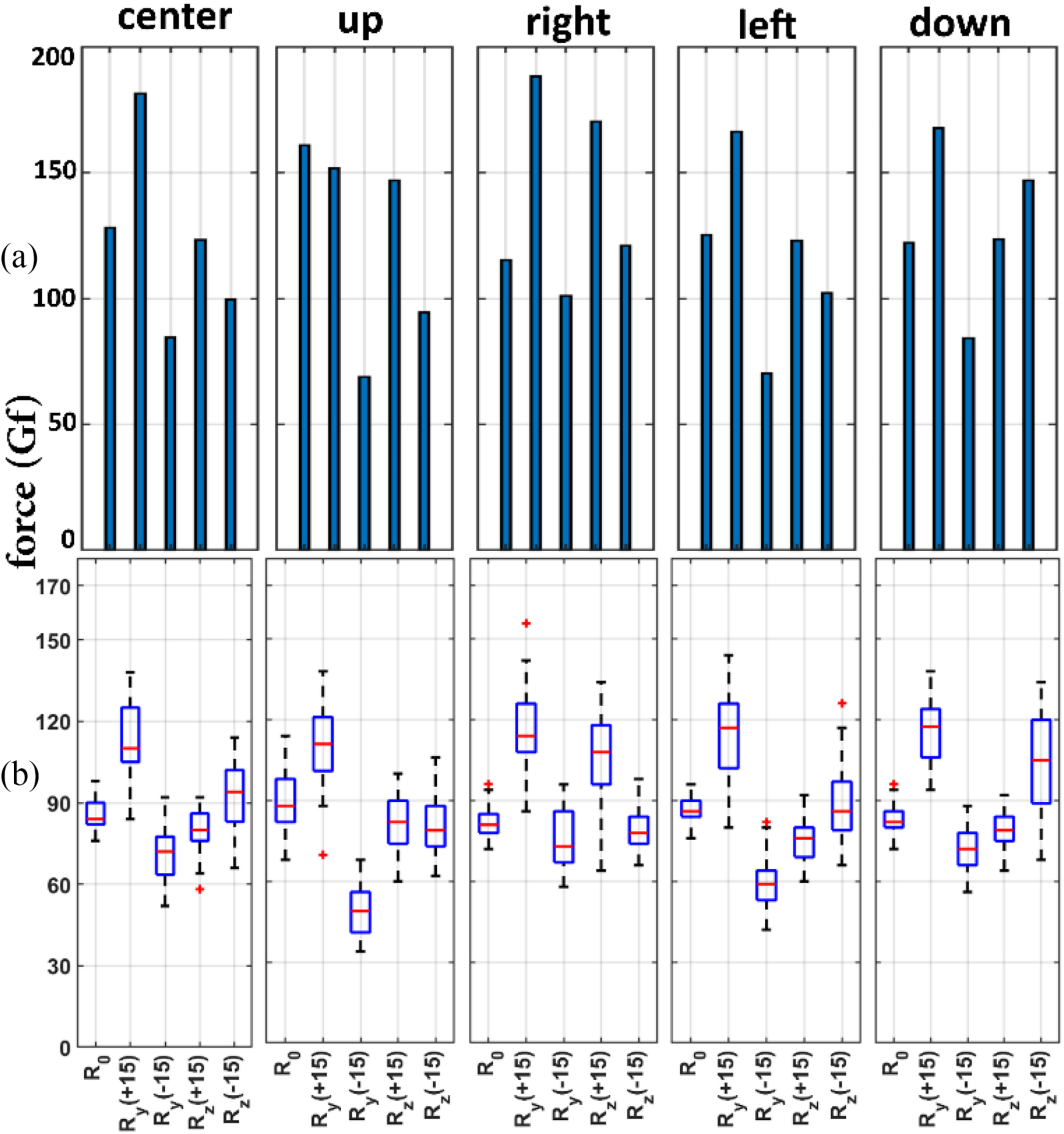
Effect of guidewire entry orientation, (a) Simulated and (b) Experimental measurements of force values at the guidewire tip in contact with the phantom cap for different insertion directions and positions. For each experimental test scenario, the results relate to forty independent experiments.

With regards to the force, the guidewire tip force values obtained through the simulations are provided in Fig. 4(a). While the minimum forces belonged to the ${R}_y$(−15°) insertion direction for all five insertion points, the maximum guidewire tip forces were for the ${R}_y$(+15°) insertion direction for all insertion points except the up insertion point, where there is a negligible offset with the maximum force at this point.

The experimental results of the forces of the guidewire tip in contact with the phantom arterial cap are shown in Fig. 4(b). Similar to the simulations there is a significant $(p < 0.0001)$ difference in guidewire tip force between ${R}_y$(+15°) and$\ {R}_y$(−15°) for each insertion point for our specific vessel phantom model. Table III ANOVA of the measured force dataset. Each column of Table III represents the significance of the guidewire insertion direction compared to the perpendicular insertion direction at the same insertion point. For example, column ${U}_0 - {U}_{y - }$ shows a significant ($p < 0.0001)$ decrease of 39.95 gf in the mean force values due to the alignment of the guidewire by ${R}_y$(−15°) compared to the perpendicular insertion at the up insertion point. As can be seen, there are statistically significant differences between the alignment of the guidewire by $\ {R}_y$(+15°), and $\ {R}_y$(−15°) with smaller p-values compared to$\ {R}_z$(+15°), and $\ {R}_z$(−15°) with larger p-values in most of the insertion scenarios.

**TABLE III:** Statistical Results of the Experimental Guidewire Tip Force for Different Insertion Positions and Orientations

	$\ {C}_0 - {C}_{y + }\ {C}_0 - {C}_{y + }\ {C}_0\ {C}_{y + }{R}_y$	$\ {C}_0 - {C}_{y - }$	$\ {C}_0 - {C}_{z + }$	$\ {C}_0 - {C}_{z - }$	$\ {U}_0 - {U}_{y + }$	$\ {U}_0 - {U}_{y - }$	$\ {U}_0 - {U}_{z + }$	$\ {U}_0 - {U}_{z - }$	$\ {R}_0 - {R}_{y + }$	$\ {R}_0 - {R}_{y - }$	$\ {R}_0 - {R}_{z + }$	$\ {R}_0 - {R}_{z - }$	$\ {L}_0 - {L}_{y + }$	$\ {L}_0 - {L}_{y - }$	$\ {L}_0 - {L}_{z + }$	$\ {L}_0 - {L}_{z - }$	$\ {D}_0 - {D}_{y + }$	$\ {D}_0 - {D}_{y - }$	$\ {D}_0 - {D}_{z + }$	$\ {D}_0 - {D}_{z - }$
p-value	<0.0001	<0.0001	0.9131	0.5680	<0.0001	<0.0001	0.2918	0.0215	<0.0001	0.3611	<0.0001	>0.9999	<0.0001	<0.0001	0.0010	>0.9999	<0.0001	0.0010	0.9961	<0.0001
Mean diff.	27.68	−15.05	−5.200	6.500	20.80	−39.95	−7.400	−9.550	35.10	−7.150	24.40	−2.600	27.95	−27.00	−11.35	2.725	32.83	−11.35	−4.000	20.05

As mentioned in Section A, there is a difference between the simulated and experimental force values that may be due to the complex, nonlinear, and unmodeled dynamics of the experimental setup, in particular due to the intense buckling of the guidewire during the additional (1.5 cm) feed after the initial impact on the plaque. However, the pattern of force delivered with respect to the direction of insertion is quite similar in both the simulated and experimental results, confirming that the orientation of the guidewire in certain directions with respect to the given anatomy of the vessel could significantly increase the value of force delivered at the occlusion site.

### C. Influence of Guidewire Entry Location-Elastic Model

**Fig. 5.**
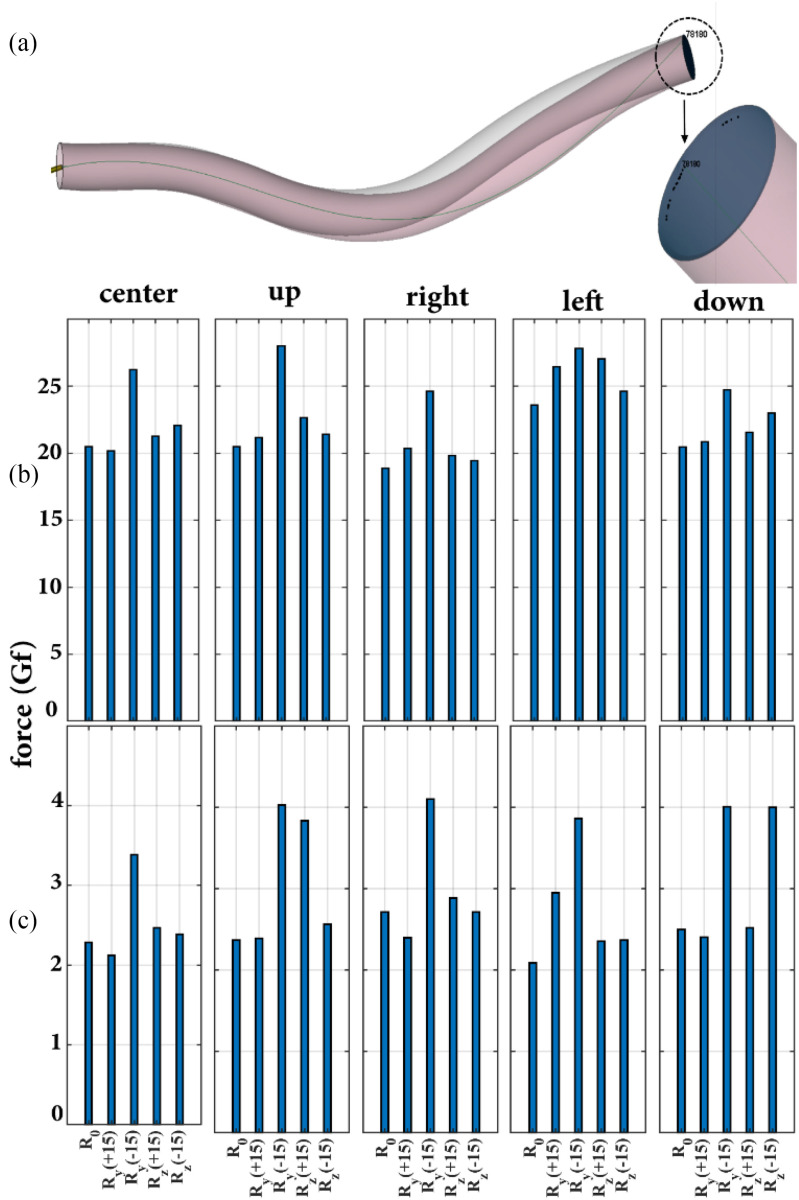
Simulated elastic artery-plaque model, (a) Position of the guidewire tip on the deformable lesion cap for different insertion scenarios. The gray and pink figures refer to the deformable artery before and after the guidewire insertion, (b) Realistic force values of the guidewire tip in contact with the deformable phantom cap for different insertion directions and positions, (c) Maximum contact force between the guidewire tip and vessel wall along the entire length of the artery for different insertion scenarios.

The reachable workspace of the guidewire tip for different insertion scenarios is shown in Fig. 5(a). As shown, similar to the rigid phantom artery-plaque model, the guidewire tip has access only to the periphery of the artery. In addition, the shape of the deformable artery before insertion and at the end of the insertion course is superimposed in a single photograph to illustrate the effects of guidewire motion on the deformation of the artery.

The magnitude of the guidewire tip force values for the simulations with a deformable artery and plaque are shown in Fig. 5(b). As expected, the range of the guidewire tip forces in Fig. 5(b) is much smaller than the corresponding values in Fig. 4(a), where a rigid artery and plaque were assumed. As shown in Fig. 5(a), the value of force delivered at the plaque site does not change significantly when only the position of the guidewire at the insertion site is changed (${R}_0$ data for different insertion sites). The maximum changes include a slight decrease of 7.9% and a moderate increase of 15% in the guidewire tip forces for the right and left insertion points, respectively vis-a-vis the center insertion point. The result obtained in this subsection for the deformable artery is consistent with the corresponding results obtained in Subsection A for the rigid artery.

### D. Influence of Guidewire Entry Orientation-Elastic Model

It is worth noting how the orientation of the guidewire at the insertion point affects the force observed at the device tip in the simulations of the deformable artery. As shown in Fig. 5(b) (${R}_{\mathrm{y}}$ and ${R}_{\mathrm{z}}$ data for different insertion points), the alignment of the guidewire at the insertion point regardless of a partic direction (y or z), not only could not decrease the force value of the guidewire tip at the site of occlusion but also increase the force value for certain directions. As observed, for this particular phantom model, the maximum forces for all insertion points refer to the case where the guidewire is oriented by$\ {R}_y$(−15°) at the insertion point. Although the interaction of the guidewire with the deformable (Section D) and rigid (Section B) artery-plaque models is different, both scenarios confirm that guidewire alignment based on the given anatomy of the vessel can increase the force of the guidewire tip at the occlusion site and thus increase the efficiency of the revascularization procedure. It is also important to consider the maximum contact force between the guidewire tip and the deformable vessel wall over the entire length of the artery. Fig. 5(c) provides this information. As shown in Fig. 5(c), the maximum force experienced by the vessel wall through the guidewire tip is related to the orientation ${R}_y$(−15°) for each insertion point. These results are consistent with those shown in Fig. 5(b), which indicate that the guidewire tip applies the maximum force value for$\ {R}_y$(−15°) orientation at each insertion point at the occlusion site. This suggests that when the guidewire is oriented around ${R}_y$(−15°) at the insertion site, the vessel is under greater tension and is therefore at greater risk of perforation.

## V. Discussion

This study was a pilot project to evaluate the feasibility of using simulation to predict the mechanical performance of a specific device, specifically its reachable workspace and deliverable forces. One limitation of the study was that the experimental results presented in Section IV were based on a rigid phantom model, while the simulations included both elastic and rigid artery models. While the simulation results for the rigid artery model were confirmed through experiments, we were not able to perform experimental validation with the deformable phantom model due to the significant challenges of creating an elastic artery phantom model that had the appropriate elasticity, friction properties, and transparency to allow for accurate device position estimation and tracking. As a result, this validation was beyond the scope of this work. However, in the future, we hope to update our results with more realistic patient-based phantom vessels that more accurately reflect the morphological properties of the tissue, including shape, structure, and viscoelastic material properties of the actual anatomy. Additionally, we envision the use of machine learning algorithms and artificial intelligence systems as a promising direction for improving PAD treatment [35], and the adoption of neural network learning algorithms to ease the burden of efficient data collection and labeling associated with machine learning algorithms.

## VI. Conclusion

In this paper, we demonstrated that it is possible to increase the efficiency of the revascularization procedure and the success rate of CTO crossing with severe calcifications for a given vessel anatomy by carefully positioning and reorienting the guidewire at the entry site in a CTO-SFA application. This information could be highly valuable to clinicians, as it allows them to potentially predict the performance of a specific device based on the patient's anatomy and better plan the procedure and select the appropriate devices.
